# The many faces of thyroxine

**DOI:** 10.3934/Neuroscience.2020002

**Published:** 2020-03-03

**Authors:** Mary B. Dratman, Joseph V. Martin

**Affiliations:** 1 Department of Medicine, University of Pennsylvania, Philadelphia, PA 19104, USA; 2 Biology Department, Center for Computational and Integrative Biology, Rutgers University, 315 Penn St., Camden, NJ 08102, USA

**Keywords:** thyronamines, nongenomic, deiodinases, non-canonical, iodothyronines

## Abstract

Hönes et al. have recently shown that *in vivo* interference with the apparatus of the nuclear receptor-mediated, gene-driven mechanism of triiodothyronine (T3) actions fails to eliminate all actions of T3. However, the investigators conducting that study provided little information regarding the mechanisms that might be responsible for conferring those implied gene-independent effects. Dratman has long ago suggested a system wherein such gene-free mechanisms might operate. Therefore, since news of that discovery was originally published in 1974, it seems appropriate to describe the progress made since then. We propose that thyroxine and triiodothyronine have many different structural properties that may confer a series of different capabilities on their functions. These conform with our proposal that a series of catecholamine analogs and their conversion to iodothyronamines, allows them to perform many of the functions that previously were attributed to nuclear receptors regulating gene expression. The actions of deiodinases and the differential distribution of iodine substituents are among the critical factors that allow catecholamine analogs to change their effects into ones that either activate their targets or block them. They do this by using two different deiodinases to vary the position of an iodide ion on the diphenylether backbones of thyroxine metabolites. A panoply of these structural features imparts major unique functional properties on the behavior of vertebrates in general and possibly on *Homo sapiens* in particular.

## 1. Introduction

In the early 1900s, Kendall [1] and Harington [2], each working independently in his own laboratory, contributed to the discovery of thyroxine as the principle agent of thyroid hormone action. Their research soon led each of them to present various versions of the proposed structure of that ubiquitous compound: thyroxine.

**Figure 1.**
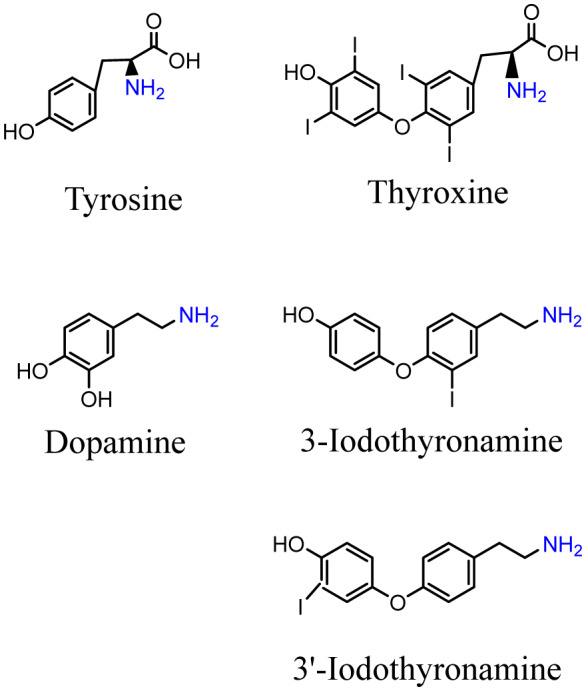
Structures of tyrosine, thyroxine, dopamine and two iodothyronamines. The N-containing groups are highlighted in blue.

Neither investigator mentioned the amino acid character of their proposed thyroxine's chemistry (see Figure 1), although it could easily have been deduced from each of the many chemical formulae of putative thyroxine molecules that they proposed and struggled to justify as the active agent of thyroid follicle function. Kendall eventually won the prize. He proposed a version of thyroxine's structure which, though later somewhat modified, became widely accepted [3].

Even as Kendall's accomplishment was being celebrated, another problem arose. Prevalence of a dogma that declared thyroxine to be inactive in the brain stood in the way of several potentially useful research efforts attempted at that time. The dogma persisted because, unlike thyroxine's effect on almost all other rodent tissues, brain tissue failed to increase its level of oxygen consumption after administration of thyroxine [4],[5]. As a result of those observations, thyroxine's action in the brain was, for a considerable period of years, ignored by investigators seeking to understand the mechanism of thyroxine's action in vertebrate organisms, especially in rodents such as rats and mice.

## 2. Early insights into the many faces of thyroxine

An early look at thyroxine's biological effects was described in a paper published many years ago (Dratman. 1974). Dratman's work as an attending M.D. in the endocrine service of the then famous Philadelphia General Hospital (PGH) had brought her in close contact with patients suffering from a variety of thyroxine-related disorders. Those experiences led her to question the widely-held belief that the adult brain is unresponsive to the action of thyroxine. Also in preparation for organizing her thoughts about these matters, Dratman made a close study of the steps taken in the course of thyroxine synthesis. Thyroxine cannot be synthesized in the absence of iodine. Other perhaps more abundant halogens might have served, but they do not lead to the formation of thyroxine or a thyroxine-like molecule. This was so, even though the element is severely deficient in many geographical regions of the earth.

It thus appeared that despite iodine's rarity, the biology of the halogen might be critical for understanding the biology of thyroxine. It turned out that one of the first things that the thyroid follicle does in preparing to synthesize thyroxine is to accumulate iodide ions from the blood stream into the thyroid follicle [6],[7].

**Figure 2.**
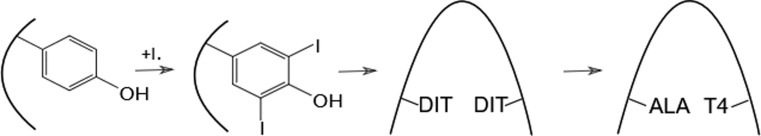
Iodination of thyroglobulin and formation of thyroxine residue. A tyrosyl residue on thyroglobulin is iodinated to diiodotyrosine (DIT). The thyroglobulin changes conformation until two DIT residues come in close proximity. A thyroxine (T4) residue is formed, leaving an alanyl (ALA) residue.

Moreover, an enzyme resident in the follicle, thyroperoxidase, is the first agent to effect a change in the nature of that collected element: It oxidizes the entering iodide ions and thus makes them capable of converting the tyrosine molecules to monoiodo- and diiodotyrosines that are among the amino acids that make up the thyroglobulin (TG) molecule [8]. The presence of those new iodotyrosine residues in TG molecules induces the refolding of TG, an action that brings together pairs of DIT residues [9]. (See Figure 2) This action, together with other small changes (such as the elimination of one of DIT's side chains) results in the assembly and incorporation of thyroxine residues into the TG protein. (See Figure 2) Thus, as the above process indicates, and the next steps demonstrate, tyrosine residues in TG are at the heart of each step taken in the process of constructing thyroxine residues in TG polymers. Moreover, incorporation of thyroxine as an amino acid residue into what were originally TG molecules, and are now outdated TG molecules (oTG) is a proteinogenic event. Although it differs from the process of proteinogenesis in mature organisms, we suggest that it may indeed represent a proteinogenic event (as it is here proposed to be) in a vertebrate fetus undergoing development.

That particular event (proteinogenesis of thyroxine in oTG molecules) is followed by an unexplained activation of a lysosomal response which results in the release of lysosomal peptidases [10],[11]. These hydrolyze the entire oTG molecule, an action that allows its amino acid residues, along with its new amino acid, thyroxine, to enter the blood stream, making available all those free amino acids to organs and tissues throughout the organism.

The hydrolysis of what were no-longer-useful molecules of TG, (after they have donated their tyrosine residues to the synthesis of molecules of iodotyrosines and thyroxine), are soon replaced with freshly synthesized TG molecules, which are now ready to use their tyrosine substituents as substrates for producing newly minted thyroxine molecules.

Although no biological explanation has been given regarding these last few steps of thyroxine synthesis, it appears that thyroxine's presence as an amino acid residue in TG induces the particular series of lysosome-directed events just described. Given that the function of lysosomes is said to be responsible for removal of unwanted material from the cell, it may be that proteinogenesis of thyroxine is seen by the thyroid follicle as an unwanted event. Therefore, when that process, proteinogenesis of thyroxine, leads to its incorporation into what had been the TG protein and is now oTG, the lysis of that protein may be following a pre-programmed destructive process which provides space for insertion of a new protein that advances the processes of development.

Is the incorporation of thyroxine as an amino acid residue into protein deleterious? Is the presence of thyroxine as an amino acid residue in any protein seen as a marker for proteolysis? Do the actions that follow the incorporation of thyroxine into the oTG protein have the ability to mark that site as one undergoing development and is awaiting the insertion of a protein that is capable of moving development forward? Finally, is the process of thyroxine proteinogenesis adding to or changing the many faces of thyroxine?

Although no firm answers were then available, we now suggest that the processes we observed during thyroxine proteinogenesis may have a role in the process of human fetal development, or even, as we will soon discuss, in the metamorphosis of amphibians. The ability of thyroxine to undergo proteinogenesis during fetal life, if reasonable and reproducible by others, and the possibility that it may, in so doing, induce destruction of certain no-longer-useful proteins in the tadpole-to-frog transition (metamorphosis), may answer many questions and provide further evidence for additional faces of thyroxine, a subject to which we will soon return.

## 3. Thyroxine and its iodothyronine derivatives are amino acid analogs of tyrosine

Although it had long been agreed that thyroxine is derived from tyrosine, it has turned out to be far more biologically insightful to recognize thyroxine as an amino acid analog of tyrosine rather than simply a derivative of tyrosine.

The tyrosine amino acid analog character of thyroxine was first described in 1974 [12]. This paper emphasized that thyroxine is an amino acid and has a close chemical analog relationship with tyrosine. As an analog of tyrosine, thyroxine can substitute for tyrosine in many chemical and physiological reactions.

Moreover, because we are considering processes that occur in fetal life, as well as in very early post-partum life, it seemed possible that important structural features (faces) of thyroxine in fetal life might be different from and therefore might cast light on thyroxine's still mainly mysterious biological functions during adulthood. We will look into this shortly.

## 4. Thyroxine and its iodothyronine metabolites are also aromatic amino acid analogs of tyrosine

Openness to the possibility that there might be many faces of thyroxine yet to be discovered soon led to recognition that thyroxine and its iodothyronine metabolites are not only *amino acid* analogs of tyrosine, they are also *aromatic* amino acid analogs of tyrosine. That identity requires the presence of at least one aromatic ring in the compound's structure. Because thyroxine and each of its many amino acid metabolites have double the number of aromatic rings required of aromatic amino acids, they are eminently qualified to be ranked in that category.

The identity of thyroxine as an aromatic amino acid also leads to its rank as “essential”, a condition that identifies aromatic amino acids as essential for life-maintenance of the organism in which they function. This is certainly true of thyroxine which is known to be essential for the persistence of vertebrate and human life (see [13]). The precursor of thyroxine, tyrosine, is derived from phenylalanine, an essential amino acid. Therefore, the lineage of both tyrosine and thyroxine conform with classification as an “essential” aromatic amino acids.

Essentiality of an amino acid is hardly a trivial attribute, given that the organism cannot long survive without it. It has been clear for some time that vertebrate organisms and their evolved descendants cannot survive without thyroxine, although they do survive for a time as impaired individuals, with many deficits, especially as regards the functions of their brains (see [13]).

Failures to provide enough iodine ions for the synthesis of sufficient amounts of thyroxine to satisfy the needs of the population have been identified in many communities throughout the world. These peoples are often located in mountainous regions of territories remote from the sea (see [13]).

Although known to be a rare element, iodine is relatively abundant in the sea. However, as the distance of residence from the sea increases, the availability of iodine decreases to the point where its supply may be less than required to maintain a healthy iodine-sufficient and thyroxine-sufficient population. Thyroxine deficiency arises when iodine is insufficient for the synthesis of the amounts of thyroxine that vertebrate life demands.

Thyroxine deficiency also leads to deficiency of certain thyroxine metabolites. Thus, in iodine-deficient populations, many thyroxine-dependent metabolites will not be produced in required amounts. Paucity of thyroxine and its metabolites may deprive the organism of a recently discovered category of adrenergic agents which are known as iodothyronamines [14].

When thyroxine molecules are freely available, their lipid nature (endowed by their two aromatic rings) makes them susceptible to the actions of decarboxylases. The latter enzymes are known to be drawn to lipid environments as preferred sites of their decarboxylating activity [15]–[17]. Decarboxylases are particularly useful for the conversion of amino acids to amines. The aromatic character of thyroxine and of its metabolites qualifies them as highly lipid substrates for decarboxylase enzymes. However, if thyroxine is scarce (as it is in iodine-deficient communities), decarboxylase enzymes may have available many fewer of its required lipid substrates. This limits those processes which are known to depend on the availability of thyroxine molecules. Amphibian metamorphosis is one such well-studied process and the normal progression of human fetal development is another. These issues are discussed in greater detail in subsequent sections of this manuscript. Thyroxine is not known to be a proteinogenic amino acid, yet we shall see that many important but unexplained events occur when incorporation of thyroxine among the protein molecules of the oTG when iodine-sufficient environments prevail. At that time the entire transformed oTG molecule is hydrolyzed by the activity of lysosome-derived peptidases. Hydrolysis of oTG by those peptidases releases its constituent amino acids (including its newly synthesized amino acid, thyroxine) into the circulation. Once in the circulation, thyroxine molecules become bound to thyroxine binding protein and are taken up into tissue cells only during the short intervals when thyroxine and those thyroxine-binding molecules are in the free state of their binding equilibrium. When in that free state, they are taken up by thyroxine transporters [18]. Upon arrival within brain cells and somatic tissue cells thyroxine encounters deiodinases.

## 5. The indispensible role of deiodinases

Deiodinase enzymes are abundant and widespread throughout the organism. They function broadly within brain cells and in somatic tissue cells. Deiodinases are found in both glial and neuronal cells of the brain and are of three types dubbed D1, D2 and D3 [19],[20]. Deiodinase action removes one iodide ion from thyroxine and iodothyronines at each active encounter with them or their partially deiodinated products according to the organism's need for those products.

Because the D2 deiodinases are located close to the cell nucleus, they are thought to devote most of the T3 they produce from T4 to use as ligands for the T3 nuclear receptor [21]. On the other hand, D1 is located on the inside of the plasma membrane and is considered a strong contributor to cytoplasmic T3 [21].

During euthyroidism, D2 and D3 each respectively produce the rival thyroxine metabolites T3 and revT3. D2 removes an iodine from the outer (phenolic) ring of T4 leading to the formation of a molecule of T3, while removal of an inner (tyrosyl) ring iodine by D3 leads to the creation of a molecule of revT3 [19],[20]. T3 is known, as a result of many experiments to be more active than thyroxine or its T3-derived iodothyronine metabolites [22]. On the other hand, the actions of revT3 are, in our view, still controversial. However, as you will see, we have made contributions to and taken sides in that controversy.

RevT3's blocking action is generally seen in reviews dealing with deiodinases as a means of ridding the immediate deiodinase environment of “excess T4 molecules” on the basis of a mistaken notion that, when T4 is converted to revT3, that product gets rid of “extra’” T4 molecules because they are no longer capable of further metabolic activity. Exactly why it is necessary to get rid of extra T4 molecules is not explained.

Nor is it factual that when thyroxine is converted to revT3, it is no longer capable of activity [23].

Instead, we point to the D3 product, revT3, which, during both euthyroidism and hyperthyroidism, acts to modulate the action of T3 molecules throughout the organism as they may respond physiologically to changes in the environment and unfavorable environmental events. Therefore, we suggest that the formation of revT3 and T3 molecules by deiodinases can allow them to function as rivals which modulate each other's local and even general effects. They thereby help to maintain adrenergic equilibrium. However, if the call for establishing equilibrium persists, and/or requires a more broad or prolonged response, the participation of the modulating rivals will persist and may extend beyond what was originally perceived as a transient stress-derived signal. In such a case, norepinephrine and epinephrine may also participate.

## 6. The importance of thyroxine sufficiency for normal fetal and early post-natal development

A great deal has been learned about the diverse actions of thyroxine simply by studying populations of healthy living humans and noting their differences from populations of individuals brought up in iodine deficient (and therefore thyroxine deficient) environments (see [13]).

Those comparisons have shown that thyroxine and its metabolites are essential participants in almost all aspects of normal human fetal growth and development and may also be required for the healthy initiation of the epochs of post-natal life such as puberty, fertility, pregnancy, gonadopause and healthy aging of human subjects.

All of these landmarks are fundamental aspects of growth and developmental biology, which are also the targets of thyroxine and its metabolites throughout fetal development. We envision fetal development as a process that of necessity involves two distinct phases in whatever organ or tissue is subject at a particular time to developmental change. The first, destructive phase, is signaled by the incorporation of a thyroxine molecule into a target protein. This is a particular and unique type of proteinogenesis of thyroxine occurring during fetal development, although it also operates at the end of thyroxine synthesis in thyroid follicles. There, after devoting its tyrosine residues to the synthesis of thyroxine, thyroglobulin, now containing a molecule of thyroxine, is attacked by peptidases that cleave TG's peptide bonds without harming its constituent amino acids, now containing a molecule of thyroxine and releases them (plus thyroxine) intact into the circulation.

The second, reconstructive phase of fetal development is devoted to introducing a set of appropriate protein structures into the space left by the destructive phase. Those replacements should be capable of performing the new functions required of this reconstructive developmental phase.

To address that issue in greater detail, it has been useful to consider the developmental processes observed in amphibian metamorphosis. *In vivo* introduction of thyroxine into the circulation of tadpoles destined to become frogs may allow a proteinogenic action of thyroxine to occur [24],[25] (as it does during thyroxine synthesis in thyroid follicles). There, a series of events follow that we propose are typical of the changes made during the early phase of fetal development. Thus, developmental events introduce the need for a destructive process that initiates the metamorphic transition. Use of the terms first and last refers to each individual metamorphosing component, as it is undergoing developmental change, and does not apply to the whole organism until metamorphosis is complete.

To make these points more coherently, we cite from two studies performed by the same group of authors [26],[27]. Their papers are highly relevant to our own independent view of developmental change in amphibian metamorphosis. The authors have chosen to study the changes that take place in the tadpole gut as influenced by thyroxine action (the exact action is not specified) during the beginning of the amphibian metamorphic transition. The first paper [27] deals with changes which occur in the “destructive phase” which we propose is the one which follows after thyroxine is incorporated into the outmoded sites, those no longer useful proteins in the tadpole destined to leave its aqueous environment. These processes make room for those new proteins which, upon synthesis, are useful to the impending adaptation to the terrestrial life of the mature frog. Those proteins are inserted into the space left by the destructive phase of fetal (or amphibian) development.

We thus propose (although the authors of the above do not suggest this) that thyroxine may be subject to proteinogenesis during the initiation of tadpole metamorphosis. There is little doubt that the program that manages this level of thyroxine proteinogenesis is likely to be quite different from the ones that occur in post-partum vertebrates. We see the initiation of the metamorphic event as one that will result from incorporation of thyroxine in the proteins destined to undergo proteolysis. This will make a space left by protein that has outlived its usefulness and that now marks a tadpole protein for lysosomal-induced destruction (as happens in the thyroid follicle after proteinogenesis of thyroxine occurring during the terminal phase of its synthesis in thyroid follicles).

The second, reconstructive phase is described in the second study made by these authors [26]. It is what the authors consider to be the response ushered in by the action of D2 which produces T3 which may enter the cell nucleus and thereby activate its nuclear receptors. The gene changes that result may produce proteins that fill the space appropriately left by destructive phase of the metamorphic process. That new space allows the insertion of frog proteins with attributes that allow them to begin the advanced second stage of metamorphosis as it stimulates genes that allow production of proteins useful to the fully metamorphosed terrestrial life of the frog.

These two phases of thyroxine-dependent developmental change closely resemble our view of what we propose takes place during both the destructive and reconstructive phases of fetal and amphibian development in the gut (and as suitable, in all the other tissues). The key word here is “development”, the gut being only an example of what we propose happens in all other tissues and at probably successive times while mediating the developmental changes that occur during amphibian metamorphosis. These are required to effect the conversion of a water-immersed tadpole into the mainly terrestrial life of the frog.

The changes undergone in gut proteins of tadpoles which have just incorporated thyroxine molecules is what occurs in most amphibia starting the process of their metamorphosis. It may be the prototype for actions of thyroxine in developing rodents, and possibly in *Homo sapiens* in utero. The second phase depends on the action of genes which respond to the actions of T3 in the nuclei of new tissues and which will activate genes that produce the proteins capable or filling the spaces left by the destructive phase.

The same authors [28] write: *the initial response to TH induction in tissues is cell proliferation. Notch is one of the few genes that is differentially expressed exclusively in the brain in the first 48h of TH induction studied in these experiments. The TH-induced gene expression changes in the tail are different from the limb and brain programs. Distinct muscle and fibroblast programs were identified in the tail. Dying muscle fibers in tail (marked by active caspase-3) up-regulate a group of genes that include proteolytic enzymes. At the climax of metamorphosis, tail muscle down-regulates more than half of the genes that encode the glycolytic enzymes in the cytoplasm*.

We see amphibian metamorphosis as the function of thyroxine which, upon incorporation into outmoded areas of protein structure, initiates the destructive phase in those tadpole tissues and then utilizes constructive mechanisms to insert the appropriate tissues needed to accommodate the needs of frogs as they emerge into the terrestrial phase of their life on earth. It does so by stripping the tadpole of its outmoded protein features and thereby makes room to substitute them with the newly developed, appropriately advanced frog proteins.

Therefore, as regards the metamorphic process, we have suggested that sites of those ready-for-destruction proteins may become activated by the presence of thyroxine, introduced by a tadpole-appropriate proteinogenic step [24] by using the incorporation of thyroxine into its no longer useful protein. Thus, the proteins which contain the newly incorporated thyroxine molecules may be marked for their readiness to be removed by lysosome-directed peptide hydrolysis, a process that attacks the thyroxine-containing protein without harming its constituent amino acids. This is referred to as the destructive phase of amphibian metamorphosis. The results of these steps taken are as follows: certain proteins in the tadpole are not useful to the frog. Therefore, they are disposed of by incorporating single molecules of thyroxine into those proteins. These metabolic steps are followed by the synthesis of proteins that function to advance the structure of the newly emerging frog functioning in a terrestrial environment.

Those last events may be mediated by introduction of T3-liganded nuclear receptor-directed genetic mechanisms. Alternatively, they may come from some other process, as intimated by the work of Hönes et al. [29]. When preceded by the destructive phase, this gene-directed constructive phase may complete the remodeling required for the advances in structure needed by a growing frog.

We suggest that the process of thyroxine proteinogenesis during amphibian development is one that differs from proteinogenesis during adult vertebrate life. The exact process that takes place during fetal life is unknown to us, nor could we find any in the older and recent literature that is helpful in that regard. We only suggest that thyroxine proteinogenesis may help to mark the proteins that identify those that are no longer useful to the developing organism and are therefore ready to enter the “destructive” phase of fetal development.

We see that phase of thyroxine action which is started by the thyroid gland of the tadpole (or addition of thyroxine to the water in which the tadpole swims). Introduction of thyroxine at the appropriate time in the life of the tadpole is known to initiate the metamorphic process. Hydrolysis of outdated proteins does not degrade the amino acids themselves and only returns them to the circulation for use in another round of developmental change.

We then asked: if thyroxine is a proteinogenic amino acid, why is it so rarely found as an amino acid residue in protein molecules? Is it that thyroxine's incorporation as an amino acid residue in a protein marks that protein for destruction? Does it do so by instructing lysosomes in the cells undergoing either fetal development or amphibian metamorphosis to release peptidases that hydrolyze that protein and thereby return its recently incorporated amino acid, thyroxine, into the free thyroxine pool?

Thoughts responsive to those questions led to the following prediction: that, starting early in vertebrate history, proteinogenic actions of thyroxine in developing organisms were not necessarily the same as those transpiring in adult organisms. Actually, they should not be the same, given that the concept of development applies to a biological phase that is changing, and not yet engrained. A similar transition is seen during the terminal phase of the process of thyroxine synthesis in thyroid follicles.

To get rid of the now-outmoded oTG proteins, a device is used that had been effective during the last steps of thyroxine synthesis. There, when thyroxine synthesis is complete, thyroxine molecules are incorporated into the oTG proteins marking those TG molecules as no longer useful for the process of thyroxine synthesis. That device conforms to the demands made by development on the old, no longer useful, proteins that need to be hydrolyzed to make room for the new. This appears to be the pattern followed for the events that follow the proteinogenesis of thyroxine in TG proteins during thyroxine synthesis in thyroid follicles. It may also serve as a device during development for removing outmoded proteins in order to make room for the new ones.

The same process may be effective in amphibian metamorphosis where the tadpole proteins must give way to the ones required for expressing the new configurations suitable for the terrestrial life of a frog. This is implied by [26], which describes the recovery phase of *Xenopus laevis* metamorphosis.

## 7. The issue of doping in an organic polymer

Doping involves the addition of an impurity in a small controlled amount to a pure semiconductor in order to change its electrical properties. Dopants have been mainly studied in polymers within inorganic systems.

Thyroxine and its many metabolites differ from tyrosine most prominently as a result of the presence of iodine substituents in their composition. Iodine present in thyroxine, plus the presence of two aromatic rings rather than one, form the basis for identifying thyroxine as an amino acid analog of tyrosine.

We came to consider the issue of doping when we observed that thyroxine or T3 are synthesized and engage with organic systems in a manner that produces profound and unexpected changes in energy transductions, particularly as seen during amphibian metamorphosis and human fetal development.

Doping first came to mind during attempts to engage in analyzing the poorly understood features of thyroxine action during amphibian metamorphosis, as well as during study of the conditions of the fetus of vertebrates.

## 8. Iodine may dope the tissues sensitive to hypothyroidism

Doping may be even more relevant to severe degrees of hypothyroidism than to those found in hyperthyroidism. The latter disorder likely exerts its major effect through providing an excess of activating thyronamines in all normal phases of development. We suggest that hypothyroidism is not associated with complete loss of T3-derived thyronamines. Therefore, in hypothyroidism, bodily tissues may not only be deprived of the actions of thyroid hormones; their major loss may be due to marked lowering or even absence of iodine's doping effects on the mixture of insufficient numbers of catecholamines and some very small amounts of thyronamines that are likely to be found even in most cases of severe hypothyroidism.

The combination of thyronamines and catecholamines described here allowed some new conclusions about thyronamine actions beyond what was originally proposed. The endogenous thyronamines discovered by Scanlan [14] showed that thyronamines are a reality and not simply a product of scientific intuition and speculation. Thoughts about the relationships prevailing in hypothyroidism led to a surmise that there must be other such relationships, particularly as relevant to the adrenergic activities found in patients with non-thyroidal illness. Based on the possibilities just cited, we now propose that deiodinase products act according to the type of deiodinases that produce their predominant precursors. T3, the activating precursor, is the product of D1 or D2 while the blocking products come from the action of D3. The difference between them is not only recognized from their actions but more accurately from examining their structural differences. A blocking agent may inhibit an already blocked target and produce an activation. However, in the case of thyronamines the structure of a thyronamine is most directly related to its inherent chemical blocking or activating potential. Therefore differences in structure may become the most reliable source of distinction between being blocked or activated by a particular thyronamine Thus a 3’T1AM structure is distinguished by the fact that it has one iodine in its phenolic ring whereas the revT3·derived 3-T1AM thyronamine has a single iodine in its tyrosyl ring (see Figure 1). that difference allows us to be accurately apprised of thyronamine's inherent nature. However, does the iodine present in a blocking thyronamine lose its abilities to exert an effect on energy transformations? This is a question that has not yet been subjected to experimental scrutiny. Doping may be even more critical to severe degrees of hypothyroidism than to those found in hyperthyroidism. The latter disorder exerts its major effect through providing an excess of activating thyronamines in all phases of development. This is supported by the idea that hypothyroidism is not associated with complete loss of T3·derived thyronamines. Therefore, in that condition bodily tissues may not only be deprived of the actions of thyroid hormones; their major loss may be due to marked lowering or even absence of iodine's doping effects on the mixture of insufficient numbers of catecholamines and some very small amounts of thyronamines that are likely to be found even in most cases of severe hypothyroidism.

## 9. The many faces of thyroid hormones: Some conclusions

The steps taken during the process of thyroxine's synthesis in thyroid follicles and detailed study of thyroxine's chemistry have promoted awareness of the many new faces of thyroxine. In particular, the structure of the thyroxine molecule has come under scrutiny because certain of its features provide possibilities for its action that may have, thus far, remained occult. Thyroxine is an amino acid, an amino acid analog of tyrosine and an aromatic amino acid. As such, it may properly considered to be an essential aromatic amino and therefore predisposed to become converted to aminergic neurotransmitters, a fate that is already known to occur. Moreover, there is also evidence that thyroxine may be a proteinogenic amino acid. The last two steps taken in assembling the thyroxine molecule in the thyroid gland suggests what may be a prototype for a biological mechanism that, if recapitulated during ontogeny, might help to explain the mechanism for thyroxine's profound effects on fetal development. Discussions of those and similar issues in the present report might serve to guide future investigations.

Also emphasized here is the uniqueness of the adrenergic systems and how they function in our everyday lives. Almost all other brain systems have their yin and yang aspects, fixated in their structure or in their intimate relationships with other brain activities. These will be explored in a separate communication devoted to important adrenergic pathologies.

Finally, new insights into the role of iodine in thyroxine and thyronamines have led to new avenues of thought about the biology of these unique and powerful iodine-bearing molecules and their importance for human biology. Of particular interest is the possibility that iodine in these organic iodocompounds may be acting as a dopant or dopant-like agent in versions of organic adrenergic compounds described in this communication.

## References

[1] Kendall EC (1919). Isolation of the iodine compound which occurs in the thyroid: First paper. J Biol Chem.

[2] Harington CR (1926). Chemistry of thyroxine: Isolation of thyroxine from the thyroid gland. Biochem J.

[3] Kendall EC (1929). Thyroxine.

[4] Fazekas JF, Graves FB, Alman RW (1951). The influence of the thyroid on cerebral metabolism. Endocrinology.

[5] Timiras PS, Woodbury DM (1956). Effect of thyroid activity on brain function and brain electrolyte distrubution in rats. Endocrinology.

[6] Dunn JT (1998). What's happening to our iodine?. J Clin Endocrinol Metab.

[7] Wolff J (1964). Transport of Iodide and Other Anions in the Thyroid Gland. Physiol Rev.

[8] Kessler J, Obinger C, Eales G (2008). Factors influencing the study of peroxidase-generated iodine species and implications for thyroglobulin synthesis. Thyroid.

[9] Dunn JT, Dunn AD (2001). Update on intrathyroidal iodine metabolism. Thyroid.

[10] Tokuyama T, Yoshinari M, Rawitch AB (1987). Digestion of thyroglobulin with purified thyroid lysosomes: preferential release of iodoamino acids. Endocrinology.

[11] Yoshinari M, Taurog A (1986). Physiological-role of thiol proteases in thyroid-hormone secretion. Acta Endocrinol.

[12] Dratman MB (1974). On the mechanism of action of thyroxin, an amino acid analog of tyrosine. J Theor Biol.

[13] Sawin CT, Braverman LE, Utiger RD (2005). The Heritage of the thyroid: A brief history. Werner and Ingbar's The Thyroid: A Fundamental and Clinical Text.

[14] Scanlan TS, Suchland KL, Hart ME (2004). 3-iodothyronamine is an endogenous and rapid-acting derivative of thyroid hormone. Nat Med.

[15] Berry MD, Juorio AV, Li XM (1996). Aromatic L-amino acid decarboxylase: a neglected and misunderstood enzyme. Neurochem Res.

[16] Lovenberg W, Weissbach H, Udenfriend S (1962). Aromatic L-amino acid decarboxylase. J Biol Chem.

[17] Axelrod J, Saavedera JM (1974). Aromatic amino acids in the brain. Ciba Found Symp.

[18] Friesema EC, Jansen J, Visser TJ (2005). Thyroid hormone transporters. Biochem Soc Trans.

[19] Gereben B, Zeold A, Dentice M (2008). Activation and inactivation of thyroid hormone by deiodinases: Local action with general consequences. Cell Mol Life Sci.

[20] Gereben B, Zavacki AM, Ribich S (2008). Cellular and molecular basis of deiodinase-regulated thyroid hormone signaling. Endocr Rev.

[21] Baqui MMA, Gereben B, Harney JW (2000). Distinct subcellular localization of transiently expressed types 1 and 2 iodothyronine deiodinases as determined by immunofluorescence confocal microscopy. Endocrinology.

[22] Michel R, Pitt-Rivers R (1957). The relative potencies of thyroxine and triiodo-thyronine analogues in vivo. Biochim Biophys Acta.

[23] Hercbergs A, Mousa SA, Davis PJ (2018). Nonthyroidal illness syndrome and thyroid hormone actions at integrin alpha v beta 3. J Clin Endocrinol Metab.

[24] Dratman MB, Richter ME, Lynch HA (1970). Incorporation of thyroxin carbon in protein fractions of Rana catesbiana tadpole nervous system, liver and tail. Endocrinology.

[25] Kozyreff V, Surks MI, Oppenheimer JH (1970). Demonstration of membrane-linked iodoprotein in hepatic microsomes following metabolism of the thyroid hormones. Endocrinology.

[26] Brown DD, Cai L, Das B (2005). Thyroid hormone controls multiple independent programs required for limb development in Xenopus laevis metamorphosis. Proc Natl Acad Sci U S A.

[27] Schreiber AM, Cai L, Brown DD (2005). Remodeling of the intestine during metamorphosis of Xenopus laevis. Proc Natl Acad Sci U S A.

[28] Das B, Cai LQ, Carter MG (2006). Gene expression changes at metamorphosis induced by thyroid hormone in Xenopus laevis tadpoles. Dev Biol.

[29] Hones GS, Rakov H, Logan J (2017). Noncanonical thyroid hormone signaling mediates cardiometabolic effects in vivo. Proc Natl Acad Sci U S A.

